# Species-Specific Glucose-6-Phosphatase Activity in the Small Intestine—Studies in Three Different Mammalian Models

**DOI:** 10.3390/ijms20205039

**Published:** 2019-10-11

**Authors:** Viola Varga, Zsófia Murányi, Anita Kurucz, Paola Marcolongo, Angelo Benedetti, Gábor Bánhegyi, Éva Margittai

**Affiliations:** 1 Institute of Translational Medicine, Semmelweis University, 1094 Budapest, Hungary; varga.viola@med.semmelweis-univ.hu (V.V.); sophie0924@freemail.hu (Z.M.); 2 Department of Medical Chemistry, Molecular Biology and Pathobiochemistry, Semmelweis University, 1094 Budapest, Hungary; kurucz.anita.andrea@gmail.com (A.K.); banhegyi@eok.sote.hu (G.B.); 3 Department of Molecular and Developmental Medicine, University of Siena, 53100 Siena, Italy; paola.marcolongo@unisi.it (P.M.); benedetti@unisi.it (A.B.); 4 Pathobiochemistry Research Group of Hungarian Academy of Sciences & Semmelweis University, 1094 Budapest, Hungary

**Keywords:** glucose-6-phosphatase, glucose-6-phosphate transporter, small intestine, endoplasmic reticulum, fructose

## Abstract

Besides the liver, which has always been considered the major source of endogenous glucose production in all post-absorptive situations, kidneys and intestines can also produce glucose in blood, particularly during fasting and under protein feeding. However, observations gained in different experimental animals have given ambiguous results concerning the presence of the glucose-6-phosphatase system in the small intestine. The aim of this study was to better define the species-related differences of this putative gluconeogenic organ in glucose homeostasis. The components of the glucose-6-phosphatase system (i.e., glucose-6-phosphate transporter and glucose-6-phosphatase itself) were analyzed in homogenates or microsomal fractions prepared from the small intestine mucosae and liver of rats, guinea pigs, and humans. Protein and mRNA levels, as well as glucose-6-phosphatase activities, were detected. The results showed that the glucose-6-phosphatase system is poorly represented in the small intestine of rats; on the other hand, significant expressions of glucose-6-phosphate transporter and of the glucose-6-phosphatase were found in the small intestine of guinea pigs and homo sapiens. The activity of the recently described fructose-6-phosphate transporter–intraluminal hexose isomerase pathway was also present in intestinal microsomes from these two species. The results demonstrate that the gluconeogenic role of the small intestine is highly species-specific and presumably dependent on feeding behavior (e.g., fructose consumption) and the actual state of metabolism.

## 1. Introduction

The glucose-6-phosphatase enzyme (G6Pase, EC 3.1.3.9) catalyzes the common terminal reaction of gluconeogenesis and glycogenolysis, i.e., the hydrolysis of glucose-6-phosphate (G6P) to glucose and inorganic phosphate (Pi). The G6Pase system is located in the endoplasmic reticulum (ER) and is composed of the phosphohydrolase G6Pase with an intraluminal active site, a G6P transporter (G6PT), and two other putative translocases for the reaction products, i.e., glucose and Pi. While G6Pase and the G6PT are well characterized at the molecular level, the transporters responsible for phosphate and glucose transport have not been unequivocally identified [1,2].

G6Pase is mainly expressed in glucogenic organs, i.e., liver and kidney, and has been extensively investigated since the fifties [1]. Three glucose-6-phosphatase isoforms have been identified in humans: Glucose-6-phosphatase-α, encoded by *G6PC*; pancreatic islet-specific glucose-6-phosphatase-related protein (IGRP), encoded by *G6PC2*; and glucose-6-phosphatase-β, encoded by the *G6PC3* gene. Though the three isoforms show a moderate amino acid sequence homology, their membrane topologies and catalytic sites are very similar. It is generally accepted that only G6PC, expressed in the liver and kidney, significantly contributes to the maintenance of the blood glucose level [3]. The liver G6PC regulates whole-body glucose homeostasis, constantly maintaining the blood glucose level, even during starvation. The kidney G6PC can also contribute to whole-body glucose turnover, up to 25% in a deep fasting status and diabetes, conditions under which the kidney works as a major gluconeogenetic site. The physiological roles of G6PC2 and G6PC3 are poorly defined. G6PC3 presumably hydrolyzes sugar phosphates other than G6P; a recent paper demonstrated that 1,5-anhydroglucitol-6-phosphate can be a substrate for the enzyme [4].

G6Pase is a non-specific enzyme; thus, it is able to hydrolyze many hexose-phosphates, e.g., mannose-6-phosphate (M6P); its high specificity is ensured by the presence of G6PT on the ER membrane. The transporter is encoded by the *SLC37A4* gene and is ubiquitously expressed in human tissues; some tissues contain a variant produced by alternative splicing [5]. G6PT has as well been implicated in phosphate transport as a phosphate/G6P antiporter [6,7]; however, other findings have not confirmed this assumption [8]. The permeabilization of the ER membrane (e.g., by the pore-forming agent alamethicin) abolishes the specificity of the system [9], allowing other substrates to enter the lumen.

Glucose transport in the ER can be mediated by various isoforms of the GLUT family, transporters represented mainly in the plasma membrane [10]. GLUT proteins are translated at the ER and reach their final destination via the secretory pathway; thus, their presence in the ER can be simply explained by their traveling along the pathway [11]. Recently, the presence of a GLUT10 protein has been reported in the ER [12]; however, its significance in glucose transport linked to the functioning of the G6Pase system has not yet been elucidated.

The role of the small intestine as a gluconeogenic organ has been debated for several decades. The components of the G6Pase system have been repeatedly observed by independent laboratories, and, according to some authors, the small intestine could have a role in regulating blood glucose levels [13,14,15], at least in specific conditions such as prolonged fasting, the inability of the liver to produce glucose [16], and a high-protein content diet [17]. However, sparse observation gained in different experimental animal models has given ambiguous results concerning the presence and activity of the G6Pase system. A recent review based on the measurement of gluconeogenic flux in the small intestine concluded that there is so far no credible evidence to support the concept that glucose can be produced by the organ [18].

The small intestine is a preferential place for fructose uptake and metabolism [19]. In contrast to earlier hypotheses, the small intestine converts dietary fructose into glucose [20]. The transformation requires ketohexokinase (fructokinase) activity. Thus, in species on a fructose-containing diet, the intestinal presence of the G6Pase system is a must. Moreover, we recently reported the presence of a novel pathway, composed of an F6P (fructose-6-phosphate) transporter and a phosphohexose isomerase, in liver microsomes that produces intraluminal G6P in the ER [21].

The aim of the present study was, first of all, to clarify species-related differences by reinvestigating the G6Pase system in the intestines of various mammals with different feeding behaviors. Therefore, we chose an omnivore with frugivore ancestors (human), an herbivore–frugivore (guinea pig) and an herbivore (rat) species for our investigation. The second aim of the project was to demonstrate the presence of the F6P transporter–phosphohexose isomerase pathway in intestinal microsomes.

## 2. Results

### 2.1. Activity of G6Pase System in the Liver and Intestinal Microsomes of Different Species

In the first set of preliminary experiments, we obtained liver microsomes from different species (human, guinea pig, and rat) already described to have a G6Pase system in the ER, and we analyzed their G6Pase activity by measuring produced glucose after the addition of the G6P substrate. Preceding all experiments, the detachment of possible membrane-associated cytosolic proteins was performed by a series of washing steps, as described in the Materials and Methods section.

Initially, we performed a kinetic analysis of G6Pase activity by measuring glucose production after the addition of the G6P substrate at different time points (Figure S1). Based on these measurements, we selected the appropriate time points to be used in our following activity measurements (5 and 30 min).

**Figure 1:**
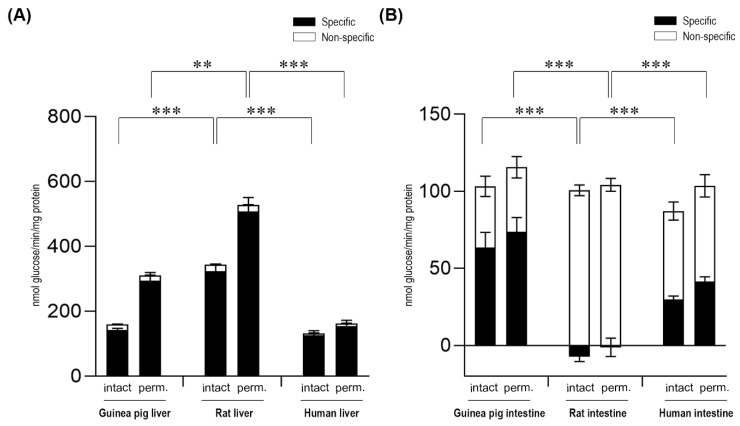
Glucose-6-phosphatase enzyme activity in the liver (**A**) and the intestine (**B**) with the glucose-6-phosphate substrate by measuring glucose production. All samples were washed three times and incubated at 37 °C in the presence of 10 mM of the glucose-6-phosphate substrate for 5 and 30 min. The figure shows the average activity measured in these time points. All experiments were performed in triplicate and repeated three times with similar results; n = 3 for all species. Bars display mean ± SEM, and statistical analysis was performed using a one-way ANOVA with a Tukey post hoc test with *p* = 0.01–0.001 ** and *p* ≤ 0.001 ***. Perm. = Permeabilized microsomes treated with the pore-forming agent alamethicin in 0.05 mg/mg microsomal protein concentration. Intact = Non-permeabilized microsomes containing intact microsomal membrane. Activity measurements were performed by the specific inactivation of G6Pase with a brief exposure of the samples to pH 5.0. Specific G6Pase activity was calculated as the difference between untreated and pH 5.0 treated samples.

After the addition of the G6P substrate, glucose production was measured in both intact and alamethicin-permeabilized microsomes. In all three species examined, we found prominent and specific G6Pase activity associated with liver microsomes (Figure 1A), which is in agreement with the role of this enzymatic system in glycemia control. The activity showed 36% and 49% latency in liver microsomes isolated from rats and guinea pigs, respectively, in accordance with other data in the literature [22]. The lower latency measured in human microsomal fractions (16%) could be attributed to the longer storage and shipment after isolation, as these microsomes were obtained commercially and were not isolated and stored in our laboratory. Detaching cytosolic proteins from microsomal membranes did not significantly affect the measured activities, although the G6P-dependent G6Pase activity slightly decreased in human samples, suggesting that in this species, the extra-microsomal dephosphorylation of the compound may also exist (data not shown). In general, the highest activity was measured in rat liver microsomes, followed by that of the guinea pig, and then human liver microsomes.

In order to clarify the unequivocal presence of the G6Pase system in the intestinal ER, microsomal fractions were isolated from the small intestine of the same species (human, guinea pig, and rat), and the G6P-dependent G6Pase activity was measured in intact and permeabilized microsomes (Figure 1B). The measurements showed a prominent inter-species variability. In intestinal microsomes of rat origin measured after the acidic pre-treatment of the samples described to inhibit G6Pase, no specific G6Pase activity was detected—the G6P hydrolytic activity was completely sustained by non-specific phosphatases. Meanwhile, in human microsomes of intestinal origin, significant specific G6Pase activity was detected, although the activity was lower compared to human liver microsomal samples. The latency of the enzyme was 27%, similar to the latency detected in human liver microsomes. Upon the addition of G6P, specific activity of G6Pase was also detected in intestinal microsomes of guinea pig origin. In guinea pig intestinal microsomes, we found a lower enzymatic latency (15%) compared to guinea pig liver microsomal samples. In both human and guinea pig intestinal microsomes, G6Pase activity was markedly decreased upon the removal of ER-attached cytosolic proteins by the applied series of washes (data not shown), which suggests that in this organ, cytosolic, non-specific phosphatases may contribute to total activity.

**Figure 2:**
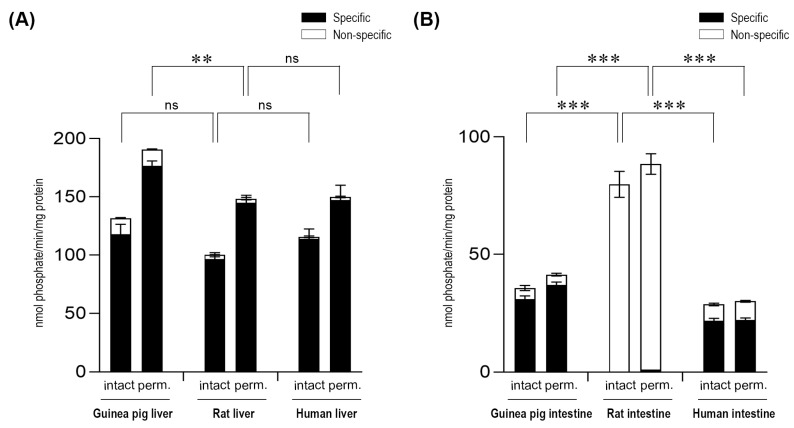
Glucose-6-phosphatase enzyme activity in the liver (**A**) and the intestine (**B**) with the glucose-6-phosphate substrate by measuring phosphate production. All samples were washed three times and incubated at 37 °C in the presence of 10 mM of the glucose-6-phosphate substrate for 10 min. All experiments were performed in triplicate and repeated three times; n = 3 for all species. Bars display mean ± SEM, and statistical analysis was performed using a one-way ANOVA with a Tukey post hoc test with the following significance levels. ns = Non-significant; *p* = 0.01−0.001 ** and *p* ≤ 0.001 ***. Perm. = Permeabilized microsomes treated with the pore-forming agent alamethicin in 0.05 mg/mg microsomal protein concentration. Intact = Non-permeabilized microsomes containing intact microsomal membranes. Activity measurements were performed by the specific inactivation of G6Pase with a brief exposure of the samples to pH 5.0. Specific G6Pase activity was calculated as the difference between untreated and pH 5.0 treated samples.

G6P-dependent G6Pase activity was also analyzed by detecting phosphate, the other end-product of G6P hydrolysis (Figure 2). Upon the measurement of the produced phosphate, similar results were obtained in both the liver (Figure 2A) and intestinal (Figure 2B) microsomal fractions, after the addition of the G6P substrate to the samples.

### 2.2. Expression of the Components of G6Pase System in Liver and Intestinal Microsomes of Different Species

**Figure 3:**
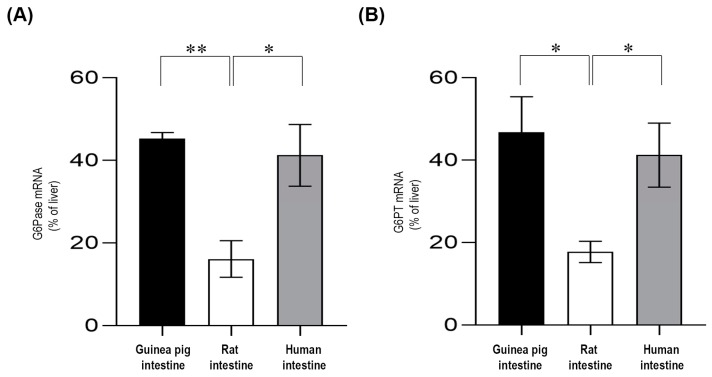
Real-time PCR analysis for the glucose-6-phosphatase (**A**) and glucose-6-phosphate transporter (**B**) mRNA expression levels. Each assay was run in triplicate, and negative controls were included; n = 3 for all species. The expression levels of intestinal mRNAs were expressed relative to the liver, which was considered 100%. Bars display mean ± SEM, and statistical analysis was performed using a one-way ANOVA with a Tukey post hoc test with *p* = 0.0–0.01 * and *p* = 0.01–0.001 **. G6Pase = Glucose-6-phosphatase; G6PT = Glucose-6-phosphate transporter.

G6Pase and G6PT mRNA expressions were assessed by real-time PCR (Figure 3). The G6Pase mRNA levels in the small intestine were calculated using the liver for comparison. Our results showed that G6Pase was constitutively expressed in the small intestine (Figure 3A), but the expression level varied according to the species. In both guinea pig and human intestinal tissue, the G6Pase mRNA levels were approximately half of those expressed in the liver. However, G6Pase mRNA expression in the small intestine from the rats showed only one-fifth of the expression of the liver. The G6PT mRNA levels in the small intestine of the three species were also investigated. It was found that the G6PT expression patterns were very similar to that of G6Pase (Figure 3B). The G6P transporter was expressed in the small intestine of guinea pig and human at approximately half of that of the liver expression, whereas the expression was significantly lower in the rat.

**Figure 4:**
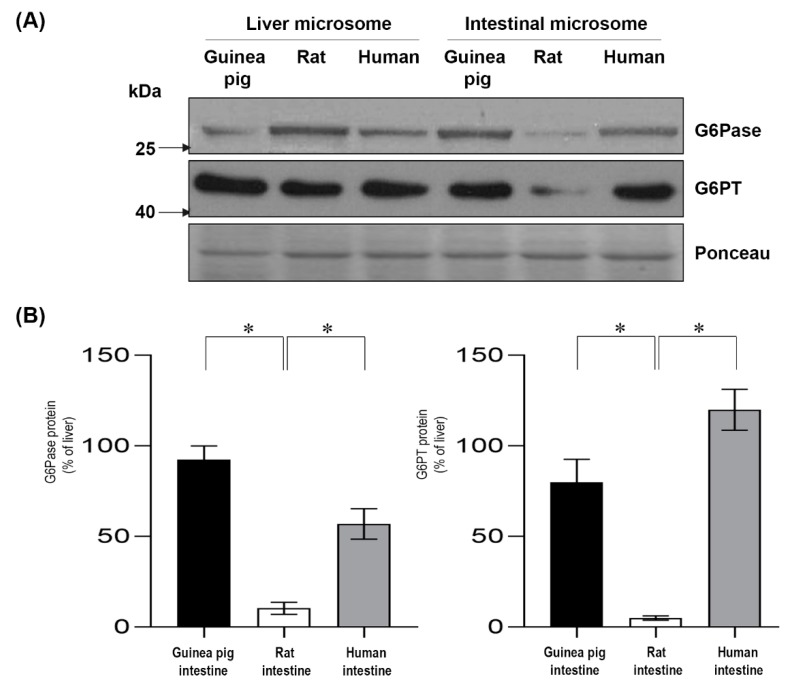
Analysis of the protein expression of glucose-6-phosphatase and glucose-6-phosphate transporter in guinea pig, rat and human intestinal microsomes. Microsomal proteins from these species were prepared for Western blot analysis (**A**). The figure shows a representative image from three separate experiments. Samples were immunoblotted with anti-glucose-6-phosphatase and anti-glucose-6-phosphate transporter antibodies. Ponceau staining was used as a loading control. Western blot images were quantified, and the expression levels of the examined intestinal proteins are shown in the percentage of liver expression (**B**). Bars display mean ± SEM, and statistical analysis was performed using a one-way ANOVA with a Tukey post hoc test with *p* = 0.05–0.01 *, *p* = 0.01–0.001 **, and *p* ≤ 0.001 ***. G6Pase = Glucose-6-phosphatase; G6PT = Glucose-6-phosphate transporter.

The expression levels of G6Pase and G6PT was also evaluated at the protein level by a Western blot analysis (Figure 4 and Figures S2 and S3). Similar to the data obtained by real-time PCR analysis, both G6Pase and G6PT were expressed in the intestinal microsomes of the guinea pigs and humans, but they were poorly expressed in the rat microsomes of intestinal origin (Figure 4A). The obtained data were quantified, and the expression levels of the liver were set as a reference corresponding to 100% (Figure 4B). In the guinea pig, the protein expression of intestinal G6Pase was similar to the liver, while the G6Pase protein level was approximately half of the expression in the liver in human intestinal microsomes. The G6PT protein levels were high in the guinea pig and human intestinal microsomes. In the intestinal microsomes isolated from rats, the expression of G6Pase and G6PT was either not detectable or very low when comparing both with the expression level of the liver or with the expression level of the intestines of guinea pig or human origin.

### 2.3. The G6Pase System of the Small Intestine Is Intraluminally Oriented

The intraluminal orientations of the intestinal guinea pig, rat, and human G6Pase systems were confirmed using the membrane impermeant substrate of the enzyme, M6P.

**Figure 5:**
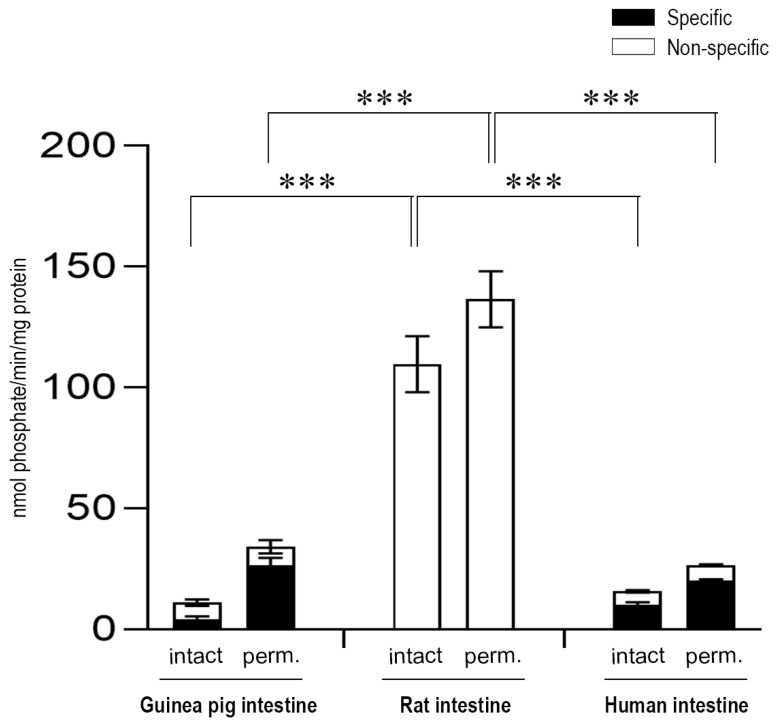
Mannose-6-phosphate hydrolyses in the intestine with the mannose-6-phosphate substrate by measuring phosphate production. All samples were washed three times and incubated at 37 °C in the presence of 10 mM of the mannose-6-phosphate substrate for 10 min. All experiments were performed in triplicate and repeated three times; n = 3 for all species. Bars display mean ± SEM, and statistical analysis was performed using a one-way ANOVA with a Tukey post hoc test with *p* ≤ 0.001 ***. M6P = Mannose-6-phosphate. Activity measurements were performed by the specific inactivation of G6Pase with a brief exposure of the samples to pH 5.0. Specific G6Pase activity was calculated as the difference between untreated and pH 5.0 treated samples.

The total phosphohydrolase activity was latent towards the non-physiological M6P substrate, which was unable to cross native membranes. The permeabilization of microsomal vesicles with the pore-forming agent alamethicin unmasked enzyme activity, which was 84% and 41% latent, respectively, in the guinea pig and human microsomes (Figure 5). These data confirm the intactness of the used microsomal membranes. The lower latency in human microsomal vesicles can possibly be explained, as these samples were commercial ones.

The data shown in Figure 5 confirm that no G6Pase activity (towards M6P) occurred in the rat microsomes of intestinal origin; i.e., M6P hydrolysis could not be detected by G6Pase that was inactivated at pH 5.0.

### 2.4. F6P-Dependent Glucose Production in Liver and Intestinal Microsomes of Different Species

It has previously been shown in liver microsomes that F6P can be transported into the ER lumen and may be subjected to yet unknown hexose-isomerase activity which generates G6P from the substrate [21]. G6P then may be hydrolyzed by the luminal G6Pase, thus providing glucose and phosphate for intraluminal reactions. G6P may also be subjected to an intraluminal hexose-6-phospahate dehydrogenase activity, which produces reducing equivalents for the reduction and activation of glucocorticoids. Thus, this direct mechanistic link between fructose consumption and prereceptor glucocorticoid activation may contribute to the development of metabolic syndromes.

**Figure 6:**
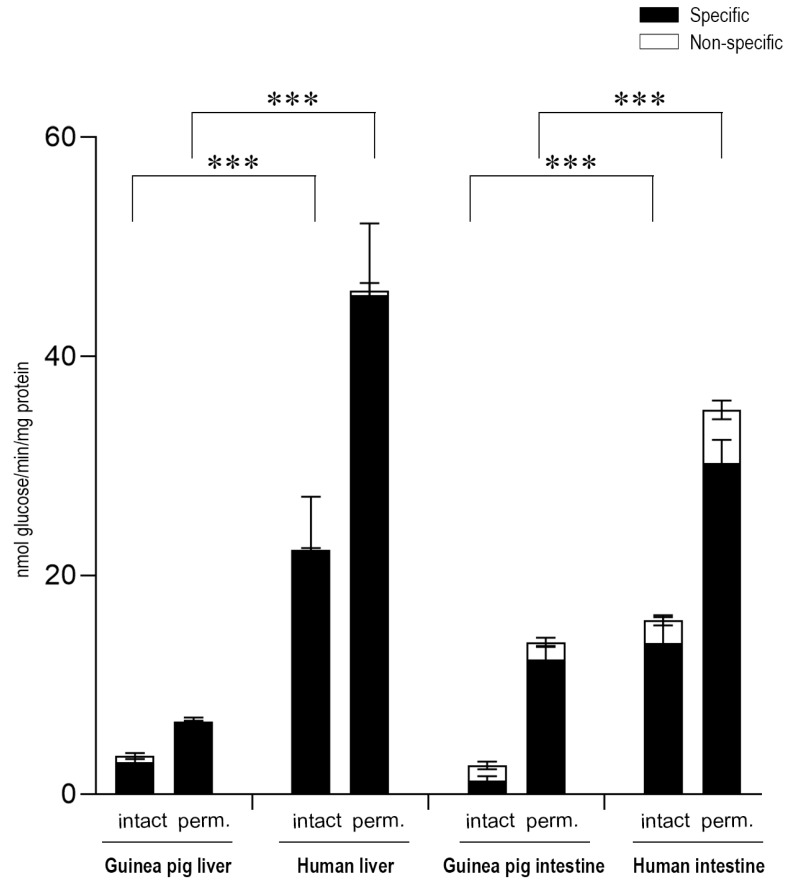
Fructose-6-phosphate-dependent glucose production in liver and intestinal microsomes. All samples were washed three times and incubated at 37 °C in the presence of 10 mM of the fructose-6-phosphate substrate for 5 and 30 min. The figure shows the average activity measured in these time points. All experiments were performed in triplicate and repeated three times; n = 3 for all species. Bars display mean ± SEM, and statistical analysis was performed using a one-way ANOVA with a Tukey post hoc test, with *p* ≤ 0.001 ***. Perm. = Permeabilized microsomes treated with the pore-forming agent alamethicin in a 0.05 mg/mg microsomal protein concentration. Intact = Non-permeabilized microsomes containing intact microsomal membrane. F6P = Fructose-6-phosphate. Activity measurements were performed by the specific inactivation of G6Pase with a brief exposure of the samples to pH 5.0. Specific G6Pase activity was calculated as the difference between untreated and pH 5.0 treated samples.

In order to clarify the presence of such a system in the small intestine, F6P was added to microsomes isolated from guinea pigs and humans, the two species in which we found an active intestinal G6Pase system; subsequently, the produced glucose was measured (Figure 6). In the first set of experiments, the glucose production from F6P was measured in liver microsomes, which confirmed previous data obtained in our laboratory [21]—a specific, latent G6Pase-dependent glucose production was measured in liver samples originating both from guinea pig and human tissues. The same measurements were repeated on intestinal microsomes. We observed prominent glucose production after the addition of F6P to intestinal microsomes of guinea pig and human origin; this glucose production was specifically derived from G6Pase activity and showed a similar latency to that observed earlier with G6P. F6P-derived glucose production was lower than that measured after G6P addition, probably suggesting a lower capacity of F6P transport or a rate-limiting nature of the isomerization step. Comparing the two species, human intestinal microsomes showed higher glucose production than guinea pig intestinal microsomes.

## 3. Discussion

The maintenance and regulation of blood glucose levels are imperative for vertebrates. In addition to that of the liver and kidney, the gluconeogenic role of the small intestine has been also considered [23]. Though many data gained in different species and experimental systems are available regarding the presence of various compounds of the G6Pase system responsible for net glucose production, the physiological function of the process is still under debate [3].

A plausible solution to the discrepancies in literature is the feeding behavior and dietary differences between the investigated species [24]. Thus, we aimed to re-investigate the G6Pase system in an omnivore with frugivore ancestors (human) [25], in an herbivore–frugivore (guinea pig) and in an herbivore (rat). We observed dramatic differences between these species that partially explain the conflicting results of the literature.

To summarize our results, we found a well detectable representation of G6Pase and G6PT, both at the mRNA and protein levels in small intestine microsomes from humans and guinea pigs. Consequently, microsomal glucose production upon G6P addition—although low compared to that of liver microsomes—was also present. The lower activity of the system in respect to liver microsomes may be attributed to the lower expression level of its components. The features of the G6Pase system (intraluminal positioning in the ER, latency, and specificity in intact vesicles) essentially mimicked the hepatic situation. In guinea pig intestine microsomes, however, we found a lower enzymatic latency (15%) compared to guinea pig liver microsomal samples, possibly suggesting a higher capacity of G6P transport or the presence of non-specific transporters in the intestinal ER. In intestine microsomes, a lower specific G6Pase activity was accompanied with higher non-specific phosphohydrolase activity that was possibly exerted by non-specific phosphatases associated with plasma membrane (brush border) of intestinal epithelial cells [26,27]. On the other hand, small intestine microsomes from rats were deprived from the elements of the G6Pase system, and no or very low glucose production—which could be attributed to the specific activity of G6Pase system—was observed upon G6P addition.

Our results demonstrated that the intestine microsomes of species possessing a functional G6Pase system are capable of glucose production upon the addition of F6P as a substrate.

A question arises: What is the background of this phenomena? Dietary effects are possible candidates for the regulation of expression. Early studies have shown that high-fructose diet induces the intestinal G6Pase system [27,28]. As a recent confirmation, it has been shown that a high-fructose diet markedly induces intestinal ChREBP-β expression [29,30]. Fructose is known to activate ChREBP transcriptional activity through increased DNA binding, phosphorylation and acetylation; for a recent review, see [19]. The intestinal protein levels of ChREBP were increased upon the addition of high-fructose diet, and this was accompanied by increased gluconeogenic gene expression [31]. ChREBP indeed regulates some genes that encode gluconeogenic enzymes (glucose-6-phosphatase catalytic subunit, G6PT, and fructose-1,6-bisphophatase) in a ChREBP*Mlx-dependent manner [32].

With respect to a putative inductive effect of fructose, it should be mentioned that a F6P transport was observed in liver microsomes; F6P transported into the ER lumen was subjected to yet unknown hexose-isomerase activity, producing luminal G6P [21]. Experiments performed in small intestine microsomes isolated from guinea pigs and humans showed that these microsomes are also equipped with a pathway like that of the liver one (Figure 6). It is tempting to suppose that this represents a new way of fructose-to-glucose conversion in the intestine and contributes to the fructose-dependent induction of intestinal gluconeogenesis in frugivores.

Another fact worth considering is the almost total absence of the system in the intestine of normal adult rats. The presence of the components of the G6Pase system and glucose production have been reported in the small intestine under different pathophysiological conditions where glucose demand is elevated (fasting, diabetes, and fetal/neonatal period) [23,33,34]. The possible role of the rat small intestine as an insulin-sensitive gluconeogenetic organ has been suggested [35]; the system is also sensitive to dietary habits [36]. However, rat intestinal glucose production is hardly detectable under normal feeding conditions in healthy species. As there are no alternative sources of glucose production, it seems that a sustained induction of the gluconeogenetic system is required in the rat intestine; returning to normal conditions results in an almost complete disappearance of the G6Pase system.

Our data are in accordance with earlier observations regarding the intestinal presence of fructose–glucose conversion in different species [37,38]. It has been described that fructose is converted to glucose in intestinal sacs in guinea pigs (and rabbits), rats lack the above mechanisms, probably due to the missing intestinal glucose-6-phosphatase system. We suggest that the presence of the glucose-6-phosphatase system and fructose–glucose conversion in the small intestine is species-specific and may be dependent on the nutritional habits of a certain species. Mammals consuming only high amount of fruits may need an effective intestinal system for using fructose as an energy source, while those covering their energy needs through plants may also lack intestinal fructose–glucose processing structures.

## 4. Materials and Methods

### 4.1. Materials

Glucose-6-phosphate (dipotassium salt), fructose-6-phosphate, mannose-6-phosphate (disodium salt) and alamethicin were from Sigma-Aldrich (St. Louis, MO, USA). All other reagents were of analytical grade.

### 4.2. Real-Time RT-PCR

Total RNA from rat and guinea pig livers and small intestines was isolated using the RNeasy Plus Mini Kit (Qiagen, Hilden, Germany), according to the manufacturer’s instructions. RNA from human tissues came from commercially available pools purchased from Ambion—Applied Biosystems (First Choice^®^ Human Total RNA Survey Panel) and from Biochain for both the liver and the small intestine. Two micrograms of RNA were reverse transcribed in a final volume of 20 μl using the Superscript^®^ III First Strand Synthesis System for RT-PCR (Invitrogen™ Thermo Fisher Scientific Inc., Waltham, MA, USA) and random hexamers. The expression levels of G6Pase and G6PT were quantified by fluorescent Real time PCR with a DNA engine thermal cycler (MJ Research, Waltham, MA, USA) equipped with the Opticon Monitor 4 software.

Analyses were performed in triplicates in a 25 μl reaction mixture. cDNA (1 μl) was amplified with Platinum SYBR Green qPCR SuperMix UDG (Invitrogen™) and 200 nM of sense and antisense primers. For guinea pig G6Pase, the oligonucleotide primers were: Sense, 5′ ACGTGATGGTCACATCTACTCTT 3‘; and antisense, 5′ AGACAGACATTCAGCTGCACA 3‘. For rat G6Pase, the oligonucleotide primers were: Sense, 5′TTCCGGTGCTTGAATGTCGT 3‘; and antisense, 5′ GCAAGGTAGATCCGGGACAG 3‘. For homo sapiens G6Pase, the oligonucleotide primers were: Sense, 5′ ACGTGATGGTCACATCTACTCTT 3‘; and antisense, 5′ AGACAGACATTCAGCTGCACA 3‘. Amplification protocol was: 95 °C (10 min), 40 cycles of 95 °C (20 s), 60 °C (20 s), and 72 °C (20 s). For guinea pig and human G6PT, the oligonucleotide primers were: Sense, 5′ TGTCCCCCTACCTGTGGGTGCTCTC 3‘; and antisense, 5′ CCAGGAGAGAGGACAGTCCGCTCTC 3‘. For rat G6PT, the oligonucleotide primers were: Sense, 5′ GGACGATTTGGGGCTCATCA 3‘; and antisense, 5‘ ACGTTGACCAGACCAACCAG 3‘. Real-time PCR was performed using the same amplification protocol described above, but the annealing temperature was 57 °C. The expression levels were referred to the liver, which was considered 100%.

The PCR amplification efficiency was calculated as reported in [39]. Each assay was run in triplicate, and negative controls (no template, template produced with no reverse transcriptase enzyme) were included. In the negative controls, no signal was detected in the investigated amplification range.

### 4.3. Preparation of Microsomal Fractions

Microsomal fractions were prepared from livers of overnight-fasted male Wistar rats (180–230 g body wt, Charles River (Europe) Laboratories Inc. Toxi-Coop Ltd., Budapest, Hungary) or guinea pigs (400–450 g body wt, LAB-ÁLL BT., Budapest, Hungary) according to a standard differential centrifugation, as previously reported [40]. Briefly, the livers were homogenized in a sucrose-HEPES buffer (0.3 M sucrose and 0.02 M HEPES, pH 7.2) with a Potter-Elvehjem homogenizer. Samples were centrifuged for 10 min at 1000× *g*, and then the supernatants were transferred and spun at 18,000× *g* for 20 min to remove mitochondrial fraction. The supernatants were then centrifuged at 195,000× *g* for 1 h to obtain the microsomal fraction. The pellet was resuspended in a MOPS–KCl buffer (100 mM KCl, 20 mM NaCl, 1 mM MgCl_2_, 20 mM MOPS, pH 7.2), and the last centrifugation was repeated with the same parameters to wash the microsomes. The pellet was resuspended in a small amount of the MOPS–KCl buffer to give a ∼30 mg/mL protein concentration.

Mucosa from the small intestine (containing mainly the jejunum and ileum) was recovered from animals (rats and guinea pigs) kept under normal conditions, having free access to nutrition, and used to prepare microsomes according to [41]. Briefly, mucosal cells were removed and suspended in a cold homogenization buffer (100 mM potassium phosphate buffer, 1 mM EDTA, 150 mM KCl, 0.1 mM dithiotreitol, 250 mM sucrose, pH 7.4) containing protease inhibitors. After centrifuging the cells three times at 3000× *g* for 6 min, the pellet was resuspended in a 4-fold volume of the homogenization buffer and sonicated for 30 sec. Samples were then centrifuged at 18,000 × *g* for 35 min. The supernatant was transferred into a new tube and was centrifuged at 195,000× *g* for 70 min. The microsomal pellet was resuspended in the MOPS–KCl buffer containing protease inhibitor cocktail (Hoffmann—La Roche Ltd., Basel, Switzerland). The microsomes were washed by another centrifugation step with the same parameters as before and were resuspended in the MOPS–KCl buffer (supplemented with protease inhibitors) to reach the final protein concentration of 2–3 mg/mL. Microsomes were immediately frozen and maintained in liquid nitrogen until use (within 3 months).

The purity of the isolated microsomal fractions was assessed by a marker-enzyme analysis, as described earlier [42]. The integrity of the microsomal membranes was determined by measuring UDP-glucuronosyltransferase activity, which showed a latency greater than 90% [43].

Microsomes from the human liver (Thermo Fisher Scientific Inc., Waltham, MA, USA) and the small intestine (Sekisui XenoTech LLC, Kansas city, KS, USA) were commercially-available pooled fractions (catalogue number of liver microsomes: HMMCPL, of intestine microsomes: H0610.I), derived from 15 (intestine) or 50 (liver) different, mixed-gender, mainly-Caucasian individual cadavers which had suddenly died due to a cerebrovascular accident, anoxia or head trauma.

### 4.4. Measurement of Protein Concentration

The protein concentration of microsomes was determined with BC A (Bicinchoninic Acid) Protein assay kit (Thermo Fisher Scientific Inc., Waltham, MA, USA) using bovine serum albumin as a standard, according to the manufacturer’s instructions.

### 4.5. Western Blot

Equal amounts of microsomal proteins were loaded onto 12% polyacrylamide gels and then blotted to polyvinylidene difluoride membranes.

Immunoblots were probed with antibodies to the G6Pase catalytic subunit and G6PT (both antibodies were used in 1: 500 dilutions; Abcam, Cambridge, UK). After reacting with the horseradish peroxidase-linked secondary antibodies (Santa Cruz Biotechnology, Heidelberg, Germany), protein bands were detected using the SuperSignal West Pico Chemiluminescent Substrate (Pierce, Rockford, IL, USA). The densitometry of the blots was performed using the ImageJ image processing software. The loading of equal amounts of proteins was also verified by densitometry analysis. The density of bands of the examined protein was normalized to the corresponding lane of the Red Ponceau-stained blot membrane.

### 4.6. Measurement of Glucose Production

Immediately before enzyme activity measurements, a series of sedimentation and buffer replacement washes was applied to remove the possible cytosolic or other contaminants loosely associated with the microsomal vesicles [21]. Briefly, microsomal suspensions were diluted in 0.5 mg/mL in a MOPS–KCl buffer containing 4.5% polyethylene glycol 8000 (wt/vol) and were centrifuged at 6000× *g* for 30 sec. After repeating the same washing step 3 times, microsomal pellets were resuspended in the MOPS–KCl buffer for subsequent assays.

Microsomes of liver and intestinal origin (0.5 mg protein/mL) were incubated in the MOPS–KCl buffer at 37 °C in the presence of 10 mM G6P, M6P, or F6P for 5 or 30 min. The reaction was stopped with heat denaturation (100 °C, 5 min). After centrifugation (20,000× *g* for 10 min at 4 °C), the glucose content of the supernatants was measured by using Glucose (GO) Assay Kit (Sigma-Aldrich, St. Louis, MO, USA) according to the manufacturer’s instructions.

During glucose production measurements, the specific inactivation of G6Pase was performed by a brief exposure of the samples to acidic pH [44]; i.e., half of the microsomal samples were brought to pH 5.0 by the addition of 1 N HCl and were incubated for 20 min at 37 °C. The pH was then neutralized to 7.2 by the addition of 1 M KHCO_3_. Specific G6Pase activity was calculated as the difference between untreated and pH 5 pre-treated samples.

Microsomes were permeabilized with the pore-forming agent alamethicin in 0.05 mg/mg microsomal protein concentration.

### 4.7. Measurement of Phosphate Production

The washed microsomes of liver and intestinal origin (0.5 mg protein/mL) were incubated in the MOPS–KCl buffer at 37 °C in the presence of 10 mM G6P or M6P for 10 min. The reaction was stopped with heat denaturation (100 °C, 5 min). After centrifugation (20,000× *g* for 10 min at 4 °C), the phosphate content of the supernatants was determined by using the Malachite Green Assay according to [45,46].

### 4.8. Statistical Analysis

Three independent measurements were performed in triplicates. Differences were expressed as the mean ± SEM. For statistical analysis, GraphPad Prism 8 software (GraphPad, La Jolla, CA, USA) was used. Statistical differences among groups were evaluated by a one-way ANOVA with a Tukey post hoc test. Differences were considered to be significant if the calculated *p*-values were *p* ≤ 0.05. Significance levels are given as: *p* = 0.05–0.01 *; *p* = 0.01–0.001 **; *p* ≤ 0.001 ***.

## Supplementary Materials

Supplementary materials can be found at https://www.mdpi.com/1422-0067/20/20/5039/s1.

## Abbreviations

ER	Endoplasmic reticulum
F6P	Fructose-6-phosphate
G6P	Glucose-6-phosphate
G6Pase	Glucose-6-phosphatase
G6PC	Glucose-6-phosphatase-α
G6PC2	Glucose-6-phosphatase-related protein
G6PC3	Glucose-6-phosphatase-β
G6PT	Glucose-6-phosphate transporter
IGRP	Glucose-6-phosphatase-related protein
M6P	Mannose-6-phosphate
Pi	Inorganic phosphate
