# Supplementary material for: Species-Specific Glucose-6-Phosphatase Activity in the Small Intestine—Studies in Three Different Mammalian Models
Source: Int J Mol Sci. 2019 Oct 11;20(20):5039. doi: 10.3390/ijms20205039 (PMC6829527; doi:10.3390/ijms20205039)
Supplement: Supplementary file 1 [file ijms-20-05039-s001.pdf]

Supplementary Material

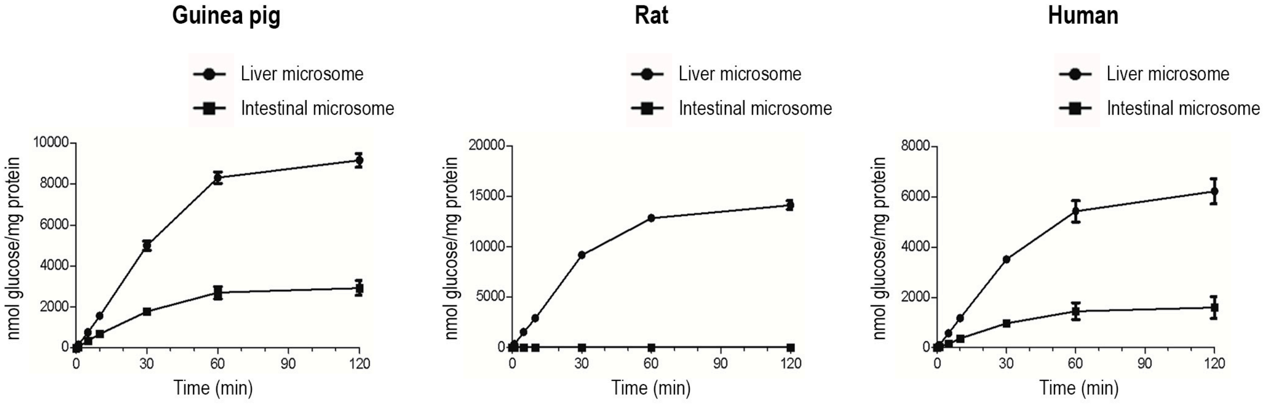

Figure S1. Kinetic analysis of glucose-6-phosphatase activity in guinea pig, rat and human liver and intestinal microsomes. Samples were washed 3 times and incubated at 37 ºC in the presence of 10 mM glucose-6-phosphate substrate for 0, 1, 5, 10, 30, 60 and 120 mins. All experiments were performed in triplicates (n = 3) for all species. The figure shows the average activity ± SD measured in these time points.

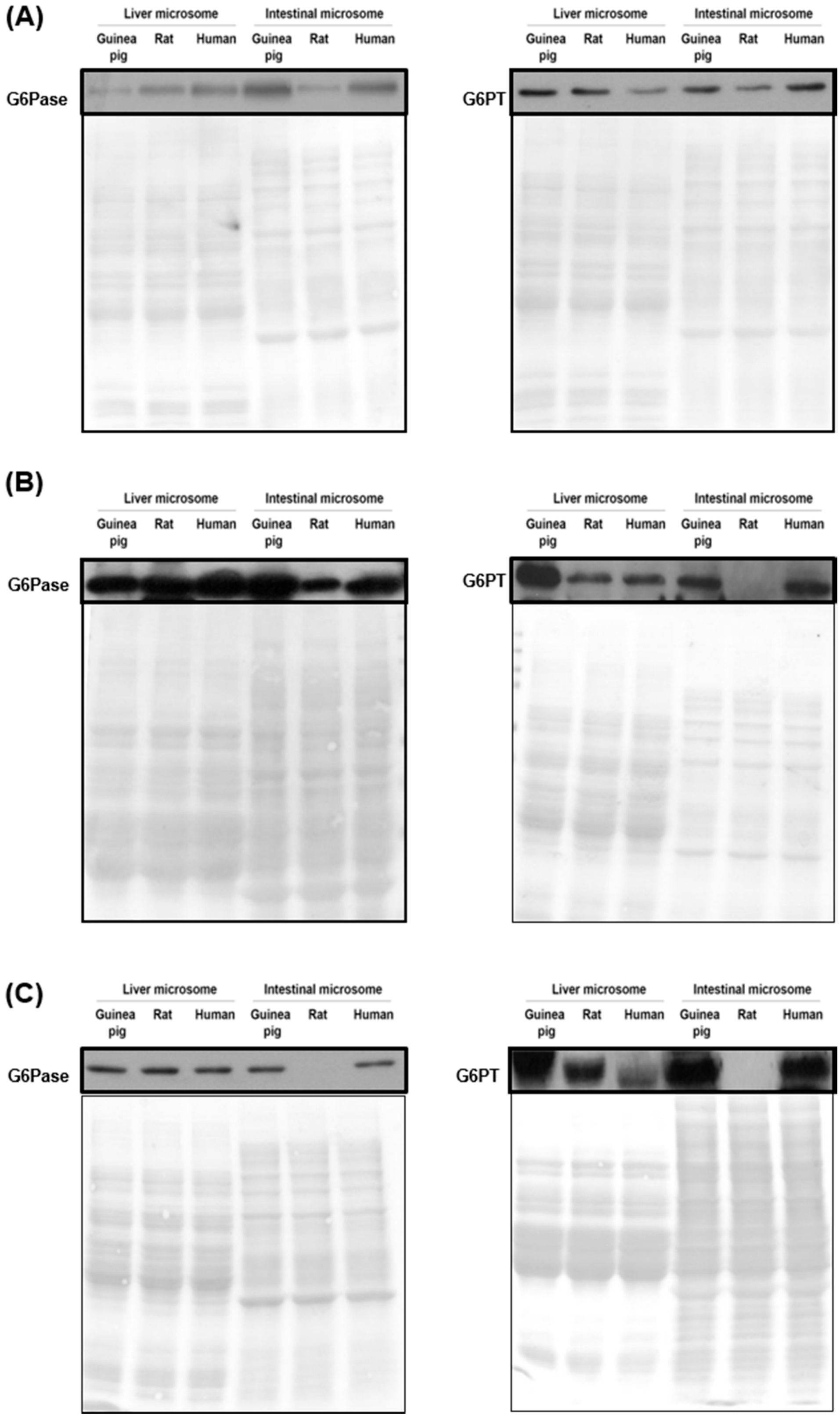

Figure S2. Analysis of protein expression of glucose-6-phosphatase (G6Pase) and glucose-6-phosphate transporter (G6PT) in guinea pig, rat and human liver and intestinal microsomes. Microsomal proteins from these species were prepared for Western blot analysis and experiments were repeated 3 times. Samples were immunoblotted with anti-glucose-6-phosphatase and anti-glucose-6-phosphate transporter antibodies. Ponceau staining was used as a loading control (density of bands of the examined protein was normalized to the corresponding lane of the Ponceau stained membrane). G6Pase, glucose-6-phosphatase; G6PT, glucose-6-phosphate transporter.

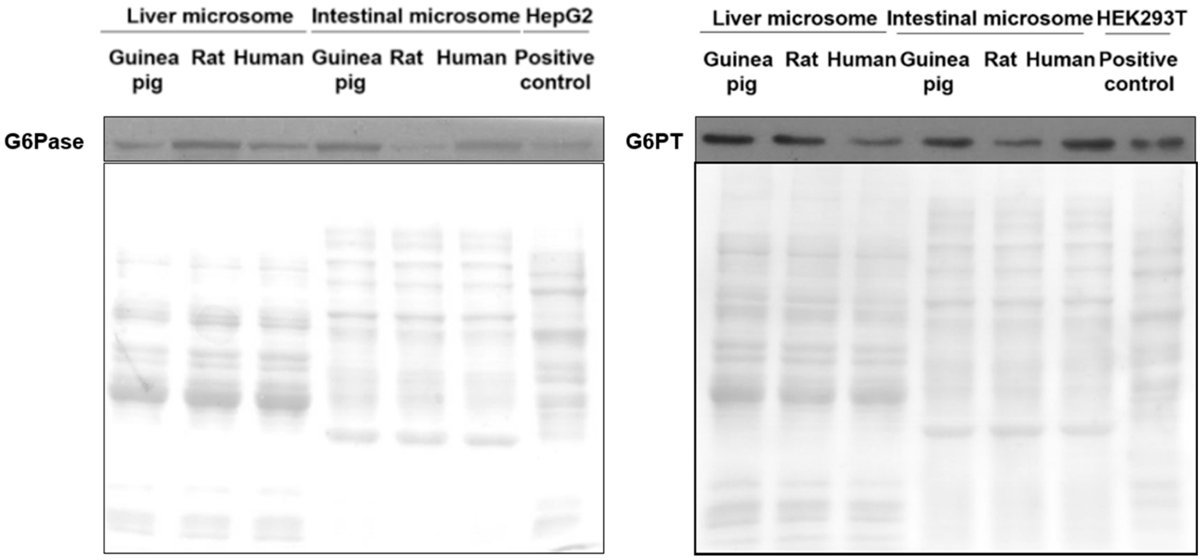

Figure S3. Analysis of protein expression of glucose-6-phosphatase (G6Pase) and glucose-6-phosphate transporter (G6PT) in guinea pig, rat and human liver and intestinal microsomes. Microsomal proteins from these species were prepared for Western blot analysis and experiments were repeated 3 times. Samples were immunoblotted with anti-glucose-6-phosphatase and anti-glucose-6-phosphate transporter antibodies. Ponceau staining was used as a loading control (density of bands of the examined protein was normalized to the corresponding lane of the Ponceau stained membrane). As a positive control, HepG2 cell lysates were used for G6Pase, and HEK293 cell lysates were used for G6PT. G6Pase, glucose-6-phosphatase; G6PT, glucose-6-phosphate transporter.
